# Digital, Crowdsourced, Multilevel Intervention to Promote HIV Testing Among Men Who Have Sex With Men: Cluster Randomized Controlled Trial

**DOI:** 10.2196/46890

**Published:** 2023-10-30

**Authors:** Yuxi Lin, Ci Ren, Meizhen Liao, Dianmin Kang, Chuanxi Li, Kedi Jiao, Lin Wang, Yu Yan, Yijun Li, Taoyu Wu, Chunxiao Cheng, Zhe Zhao, Zece Xu, Weiming Tang, Joseph D Tucker, Wei Ma

**Affiliations:** 1 Department of Epidemiology, School of Public Health Cheeloo College of Medicine Shandong University Jinan China; 2 Cheeloo College of Medicine Shandong University Jinan China; 3 Institution for AIDS/STD Control and Prevention Shandong Center for Disease Control and Prevention Jinan China; 4 University of North Carolina Chapel Hill Project-China Guangzhou China; 5 Clinical Research Department, Faculty of Infectious and Tropical Diseases London School of Hygiene and Tropical Medicine London United Kingdom

**Keywords:** men who have sex with men, HIV testing, digital intervention, multilevel intervention, cluster randomized controlled trial, China

## Abstract

**Background:**

Despite great efforts in HIV prevention worldwide, HIV testing uptake among men who have sex with men (MSM) remains suboptimal. The effectiveness of digital, crowdsourced, multilevel interventions in improving HIV testing is still unclear.

**Objective:**

The aim of this study was to evaluate the effect of a digital, crowdsourced, multilevel intervention in improving HIV testing uptake among MSM in China.

**Methods:**

We conducted a 2-arm cluster randomized controlled trial among MSM in 11 cities in Shandong province, China, from August 2019 to April 2020. Participants were men who were HIV seronegative or had unknown serum status, had anal sex with a man in the past 12 months, and had not been tested for HIV in the past 3 months. Participants were recruited through a gay dating app and community-based organizations from preselected cities; these cities were matched into 5 blocks (2 clusters per block) and further randomly assigned (1:1) to receive a digital, crowdsourced, multilevel intervention (intervention arm) or routine intervention (control arm). The digital multilevel intervention was developed through crowdsourced open calls tailored for MSM, consisting of digital intervention images and videos, the strategy of providing HIV self-testing services through digital tools, and peer-moderated discussion within WeChat groups. The primary outcome was self-reported HIV testing uptake in the previous 3 months. An intention-to-treat approach was used to examine the cluster-level effect of the intervention in the 12-month study period using generalized linear mixed models and the individual-level effect using linear mixed models.

**Results:**

A total of 935 MSM were enrolled (404 intervention participants and 531 controls); 751 participants (80.3%) completed at least one follow-up survey. Most participants were younger than 30 years (n=601, 64.3%), single (n=681, 72.8%), had a college degree or higher (n=629, 67.3%), and had an HIV testing history (n=785, 84%). Overall, the proportion of testing for HIV in the past 3 months at the 3-, 6-, 9-, and 12-month follow-ups was higher in the intervention arm (139/279, 49.8%; 148/266, 55.6%; 189/263, 71.9%; and 171/266, 64.3%, respectively) than the control arm (183/418, 43.8%; 178/408, 43.6%; 206/403, 51.1%; and 182/397, 48.4%, respectively), with statistically significant differences at the 6-, 9-, and 12-month follow-ups. At the cluster level, the proportion of participants who had tested for HIV increased 11.62% (95% CI 0.74%-22.5%; *P*=.04) with the intervention. At the individual level, participants in the intervention arm had 69% higher odds for testing for HIV in the past 3 months compared with control participants, but the result was not statistically significant (risk ratio 1.69, 95% CI 0.87-3.27; *P*=.11).

**Conclusions:**

The intervention effectively improved HIV testing uptake among Chinese MSM. Our findings highlight that digital, crowdsourced, multilevel interventions should be made more widely available for HIV prevention and other public health issues.

**Trial Registration:**

Chinese Clinical Trial Registry ChiCTR1900024350; http://www.chictr.org.cn/showproj.aspx?proj=36718.

**International Registered Report Identifier (IRRID):**

RR2-10.1186/s13063-020-04860-8

## Introduction

Men who have sex with men (MSM) are considered one of the key populations for HIV infection worldwide [1]. A key strategy for HIV control is frequent testing of high risk individuals [2], which is the first step in the HIV prevention and treatment cascade. In China, the proportion of all newly diagnosed infections attributed to male-male sexual contact increased from near zero in 2005 to 25% in 2020 [3,4]. Although a variety of HIV testing promotion programs have been organized for MSM, the HIV testing proportion is suboptimal in Chinese MSM [5]. The high burden of HIV infection and low rate of HIV testing indicate an urgent need for effective interventions expanding HIV testing coverage among MSM. Digital multilevel interventions combined with crowdsourcing may be a promising tool.

The digital approach is defined by the use of social media, mobile phone apps, the Internet, or other technologies that use digital connections [6,7]; this approach is increasingly common in health intervention development, promotion, and dissemination [8]. In 2019, the World Health Organization (WHO) released evidence-based guidelines on digital health interventions [9]. An expanding evidence base suggests that digital health interventions are effective in HIV prevention and treatment among MSM, decreasing high-risk sexual behaviors and increasing antiretroviral treatment adherence [10-13]. Stigma and discrimination toward MSM are still prevalent in China [3]. Due to convenience and confidentiality, digital health interventions may be effective in improving HIV testing coverage among MSM while allowing them to access HIV information and services anonymously and without being judged [14-17].

Among MSM, factors limiting uptake of HIV testing are present both at the individual level (eg, a lack of awareness and knowledge of HIV testing services and HIV-related and sexual minority stigma) and community level (eg, a lack of support for HIV testing) [18]. The socioecological perspective emphasizes the interaction between, and interdependence of, factors within and across all levels of a health problem and points out that health promotion programs are more effective when they consider multiple levels of influence on health problems [19]. This theory indicates the potential feasibility of implementing multilevel interventions to address these barriers related to HIV testing among MSM. A pilot randomized trial targeting MSM living with HIV in India showed the feasibility and potential effectiveness of a multilevel intervention in decreasing sexual risk [20]. However, there remains a dearth of multilevel interventions targeting HIV testing among MSM in China.

Given the importance of the digital approach and multilevel interventions in improving health, we developed a digital multilevel HIV testing promotion intervention using a crowdsourced approach. Crowdsourcing is defined as the process of aggregating a group of nonexperts and experts to solve a problem [21] and is increasingly used in developing intervention materials or strategies tailored to meet the needs of key populations. For example, previous studies developed crowdsourced interventions to improve health behaviors including HIV testing, hepatitis C testing, and community engagement in health campaigns among MSM [22-24]. Although the effectiveness of the crowdsourced method has been shown, the feasibility and effectiveness of digital multilevel interventions for HIV testing promotion developed through the crowdsourced method are still unclear. Therefore, the purpose of this cluster randomized controlled trial was to evaluate the effect of a digital, crowdsourced, multilevel intervention in improving HIV testing uptake among MSM in China.

## Methods

### Study Design, Setting, and Participants

This study was a 2-arm cluster randomized controlled trial; the trial protocol provides more details [25]. The Consolidated Standards of Reporting Trials (CONSORT) checklist can be found in Multimedia Appendix 1. This trial was clustered at the city level. Cities were eligible if they had an MSM sentinel surveillance site and had the capacity to recruit sufficient MSM. We first selected 10 cities from Shandong province, China, including Jinan, Weifang, Zibo, Jining, Liaocheng, Qingdao, Dezhou, Weihai, Binzhou, and Heze. Since Heze did not recruit enough MSM, we included the neighboring city Zaozhuang and regarded it as part of the same cluster (Multimedia Appendix 2).

Participants were eligible if they were born biologically male, were aged 18 years or older, currently lived in and planned to remain living in 1 of the 11 cities for the 12 months after enrollment, were HIV seronegative or had unknown serum status, had anal sex with a man in the past 12 months, had not been tested for HIV in the past 3 months, and provided informed consent. Participants were recruited by sending a link to the questionnaire to potential participants through Blued (a popular social app among Chinese MSM) and community-based organizations (CBOs) in each city. Additionally, eligible participants were invited to recommend at most 5 MSM in their city to participate in this study from their social networks.

### Randomization and Masking

The details of randomization are shown in Multimedia Appendix 3. According to the city’s number of people living with HIV in 2018, average GDP in 2017, cumulative number of HIV-positive people from 2012 to 2018, and population at the end of 2017, the cities with the most similar contexts were stratified into the same blocks. Ten clusters were stratified to 5 blocks (2 clusters per block). Then, 2 clusters in each block were randomly allocated 1:1 to either the intervention arm or the control arm with random numbers generated using SAS (version 9.4; SAS Institute) software. Participants and data analysts were blinded to the intervention assignment.

### Intervention

**Figure 1:**
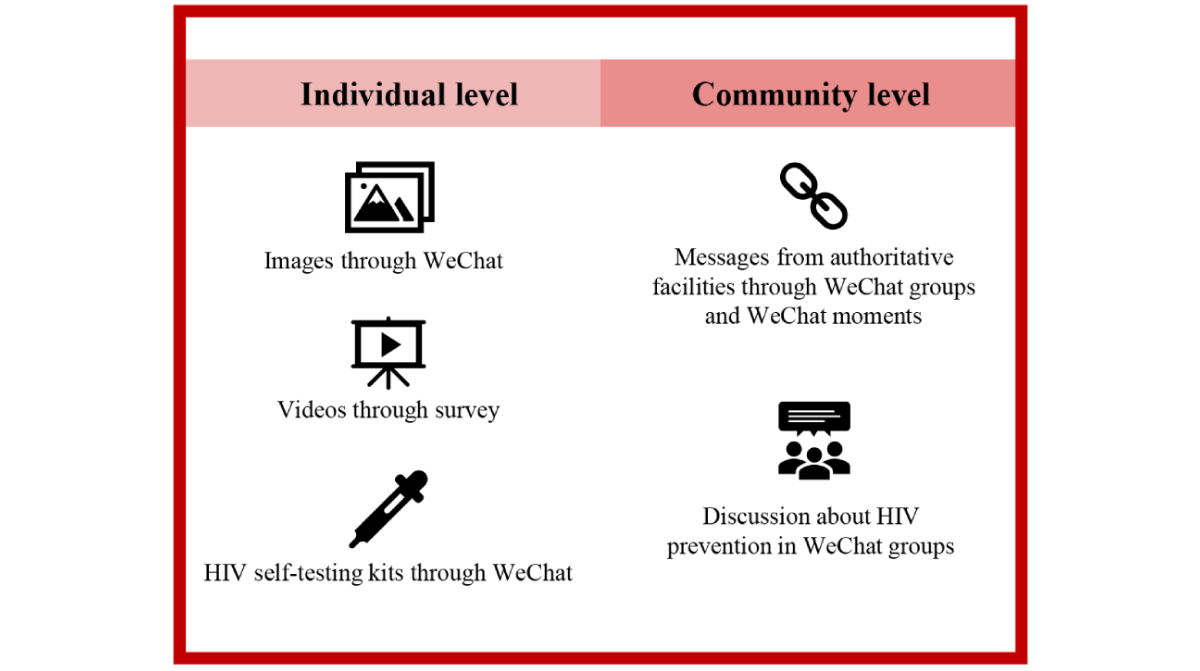
Profile of the digital, crowdsourced, multilevel intervention.

Participants in the intervention arm received a digital, crowdsourced, multilevel intervention developed through a series of crowdsourcing open calls tailored for MSM (Figure 1). Participants in the control arm received conventional intervention by their local center for disease control and prevention (CDC), including outreach activities and HIV testing services. All concomitant care was acceptable, with no restrictions on what participants may seek. The schedule of interventions across arms is shown in Multimedia Appendix 4.

The individual-level intervention included the digital intervention materials and HIV self-testing services delivered with digital tools. The digital intervention materials included 25 images and 4 videos, which were distributed by WeChat. After completing the 6-month follow-up survey, participants in the intervention arm could apply for the digital HIV self-testing services, including the free HIV blood self-test kits, use instructions, and counseling services, through WeChat. HIV self-testing counseling services were provided based on guidelines from the WHO, including pre-and posttest counseling [26]. The details of the counseling services can be found in Multimedia Appendix 4. Before testing, the staff explained the importance of regular testing and provided instructions on use of the kits. Participants who applied for and used the kits were reminded to return photos of the test results. Then, the staff helped them to properly interpret the meaning and implications of the results and provided counseling on safe sexual behaviors and regular testing for participants with negative results and referral services on confirmatory testing and further care for participants with positive results. After the 12-month follow-up, we also provided participants in the control arm with the opportunity to obtain the self-test kits.

For the community-level intervention, we set up 8 peer-moderated WeChat discussion groups in the intervention arm. All participants in the intervention arm received an invitation to join a group and made their own decision on whether to join. Each WeChat group consisted of 1 team member, 1 CBO volunteer, and about 20 participants. The messages about HIV testing and safe sexual behavior came from authoritative facilities (eg, CDCs, CBOs, and hospitals) and were shared through WeChat groups and WeChat moments (a function on WeChat that allows people to share their life with friends) biweekly. In addition, members in the WeChat groups were encouraged to discuss HIV prevention.

### Data Collection and Measurements

After the first intervention, follow-up surveys were conducted every 3 months for 12 months. Questionnaires were built using Sojump Survey Software to collect data. Demographic characteristics were collected at baseline. Sexual behaviors, HIV testing uptake, anticipated HIV stigma, HIV testing social norms, and HIV testing self-efficacy were assessed at baseline and at 4 follow-up visits, at 3, 6, 9, and 12 months. The anticipated HIV stigma, HIV testing social norms, and HIV testing self-efficacy were measured by Likert scales.

### Outcomes

The primary outcome was self-reported HIV testing uptake in the previous 3 months measured in each follow-up survey. The secondary outcomes included self-reported facility-based HIV testing uptake, HIV self-testing uptake, condomless sex behavior, social media engagement, anticipated HIV stigma, HIV testing social norms, and HIV testing self-efficacy in each follow-up survey.

### Statistical Analyses

Data from July 30, 2021, to April 28, 2022, were analyzed. A sample of 500 MSM from the 10 clusters (50 from each cluster), assuming a 30% dropout rate and an intracluster correlation of .020 (usually between .010 to .030) within 10 clusters, was planned to provide 85% power at 2-sided significance level of α=.05 to detect a 20% difference in the primary outcome. Descriptive analysis was used to describe baseline demographic characteristics for the total sample and by the intervention condition. The cumulative HIV testing uptake, incident testers (the first HIV test during the study period), HIV seroconversion, and HIV self-testing kit use were summarized. We assessed the difference in the proportion of HIV testing between study arms with 2-tailed *χ*^2^ tests at each follow-up. The intention-to-treat approach was used to examine the cluster-level and individual-level effects of the intervention in the 12-month study period. The cluster-level effect was defined as the difference in the proportion of participants who tested for HIV in the previous 3 months between intervention cities and control cities. The individual-level effect was defined as the difference in the probability of participants having tested for HIV in the past 3 months between the intervention arm and control arm. Specifically, we fitted linear mixed models (LMMs) to investigate the cluster-level effect of the intervention, in which the HIV testing proportion of each cluster across the 4 follow-ups was used as the outcome, intervention status and time were considered as fixed effects, and the sites considered as random effects. To investigate the individual-level effect of the intervention, we used generalized linear mixed models (GLMMs) to evaluate the difference in probability of receiving HIV testing in the intervention arm and in the control arm, in which the test status of every participant across the 4 follow-ups was used as the outcome, intervention status and time were considered as fixed effects, and sites and individual participants were considered as random effects. The estimated intervention effects (risk ratio [RR] for binary outcomes and mean difference for continuous outcomes) are reported with the 95% CI and *P* value.

For the sensitivity analysis, we conducted a per-protocol analysis including only participants who self-reported that they had viewed the intervention materials or had participated in our WeChat groups. For the prespecified analysis, we tested the interaction effect of the intervention between the individual level and community level. After the 6-month follow-up, we evaluated the effectiveness of the digital materials using data collected from the 3-month and 6-month follow-ups, and we evaluated the effectiveness of providing digital materials plus self-test kits using data collected from the 9-month and 12-month follow-ups. We also evaluated the effect of every component of the overall intervention, including viewing intervention images, viewing intervention videos, receiving HIV self-testing kits from the study team, and participating in the WeChat groups. In addition, we performed a subgroup analysis based on age (<30 years vs ≥30 years). We compared the demographic characteristics between participants who completed the last follow-up survey or seroconverted and participants who were lost to follow-up, and we adjusted the factors that differed between them in the analysis evaluating the intervention effect. We also used multiple imputations by chained equations and 30 imputed data sets to impute missing HIV testing data at each follow-up period and examined the intervention effect. We conducted an intention-to-treat analysis to evaluate the secondary outcomes using GLMMs or LMMs. We performed all analyses with SAS.

### Ethics Approval

This trial was approved by the institutional review board at the School of Public Health, Shandong University (20190210) and was registered in the Chinese Clinical Trial Registry (ChiCTR1900024350). All study participants provided informed consent at the baseline survey.

## Results

### Study Participants

**Figure 2:**
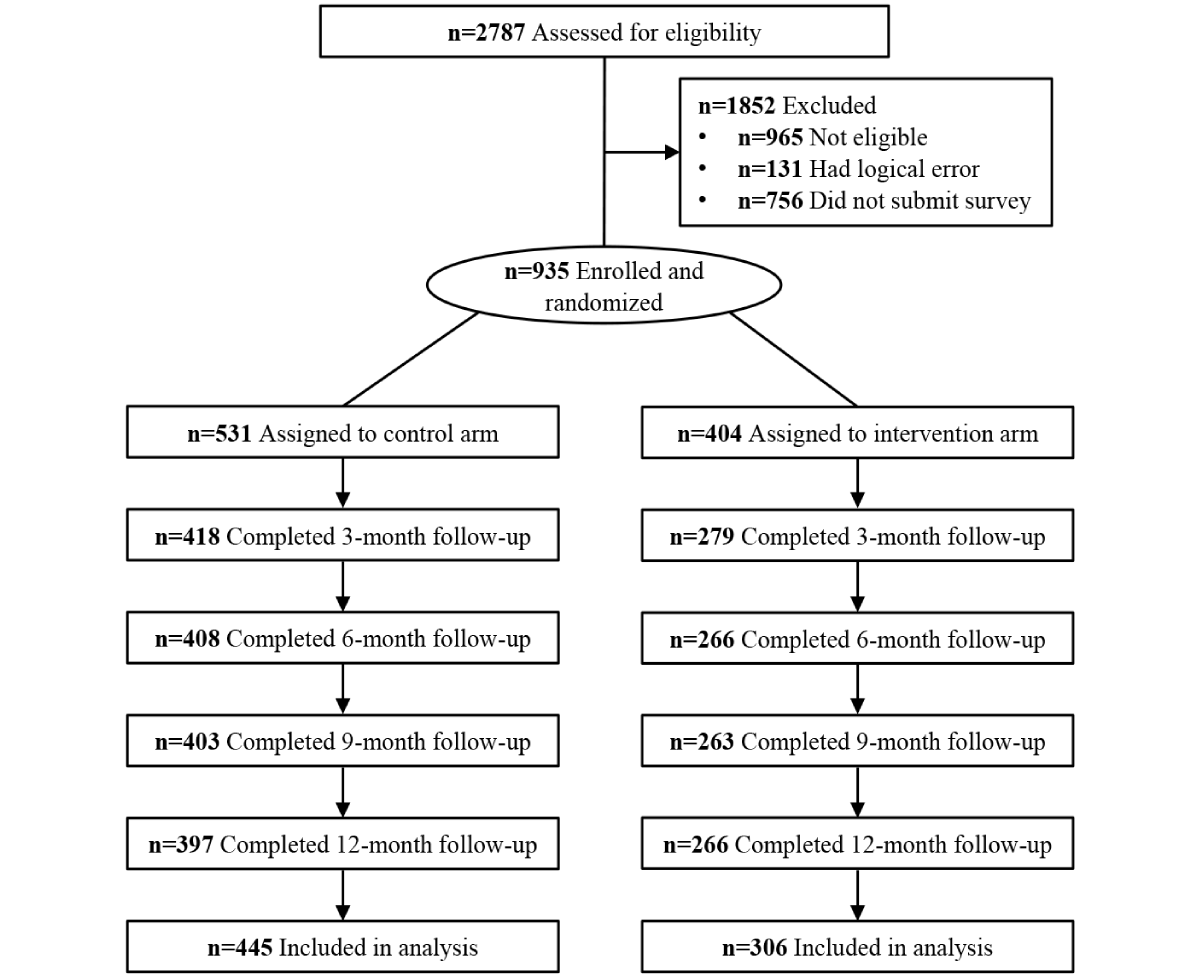
Trial profile. The control arm had 5 cities: Jinan, Weifang, Zibo, Jining, and Liaocheng. The intervention arm had 6 cities: Qingdao, Dezhou, Weihai, Binzhou, Heze, and Zaozhuang. Heze and Zaozhuang are in the same cluster.

Participants were recruited from August 6, 2019, to January 25, 2020, and followed until the last participant completed the 12-month follow-up survey on April 8, 2021. A total of 935 participants were enrolled, including 404 in the intervention arm and 531 in the control arm (Figure 2, Multimedia Appendix 5). Most participants were younger than 30 years (n=601, 64.3%), were never married (n=681, 72.8%), had a college degree or higher (n=629, 67.3%), identified as homosexual (n=668, 71.4%), and disclosed their sexual orientation to others (n=606, 64.8%). One-third of participants had engaged in condomless sex (n=314, 33.6%) in the past 3 months, and 785 participants (84%) had an HIV testing history (Table 1). After the 9-month follow-up, 10 participants HIV seroconverted (Multimedia Appendix 6). Among the 925 HIV-negative or unknown participants after the 9-month follow-up, 262 (28.3%) did not complete the last follow-up survey (Multimedia Appendix 7). The participants who completed the last follow-up survey or seroconverted differed in educational attainment and sexual orientation compared to participants who were lost to follow-up (Multimedia Appendix 8).

**Table 1:** Baseline characteristics of participants in a cluster randomized controlled trial among men who have sex with men in Shandong province, China in 2019 to 2020.

Characteristic		Total (n=935)	Control (n*=*531)	Intervention (n=404)
Age (years), median (IQR)		26.0 (23.0-32.0)	27.0 (23.0-32.0)	26.0 (22.3-32.0)
**Age group (years), n (%)**				
	<30	601 (64.3)	344 (64.8)	257 (63.6)
	≥30	334 (35.7)	187 (35.2)	147 (36.4)
**Time living in city, n (%)**				
	≤2 years	79 (8.4)	41 (7.7)	38 (9.4)
	>2 years	856 (91.6)	490 (92.3)	366 (90.6)
**Marital status, n (%)**				
	Never married	681 (72.8)	400 (75.3)	281 (69.6)
	Married	172 (18.4)	88 (16.6)	84 (20.8)
	Divorced or widowed	82 (8.8)	43 (8.1)	39 (9.7)
**Monthly income, US $^a^, n (%)**				
	<250	143 (15.3)	84 (15.8)	59 (14.6)
	251-500	139 (14.9)	71 (13.4)	68 (16.8)
	501-800	441 (47.2)	250 (47.1)	191 (47.3)
	801-1250	161 (17.2)	99 (18.6)	62 (15.3)
	>1250	51 (5.4)	27 (5.1)	24 (5.9)
**Education, n (%)**				
	High school or lower	306 (32.7)	163 (30.7)	143 (35.4)
	College or higher	629 (67.3)	368 (69.3)	261 (64.6)
**Sexual orientation, n (%)**				
	Homosexual	668 (71.4)	393 (74)	275 (68.1)
	Other	267 (28.6)	138 (26)	129 (31.9)
**Disclosure of sexual orientation, n (%)**				
	Not disclosed to others	329 (35.2)	183 (34.5)	146 (36.1)
	Disclosed to others^b^	606 (64.8)	348 (65.5)	258 (63.9)
**Condomless sex^c^, n (%)**				
	No	621 (66.4)	367 (69.1)	254 (62.9)
	Yes	314 (33.6)	164 (30.9)	150 (37.1)
**Ever tested for HIV, n (%)**				
	No	150 (16)	75 (14.1)	75 (18.6)
	Yes	785 (84)	456 (85.9)	329 (81.4)

^a^1 US $=6 CNY in 2021.

^b^Has told anyone (except sexual partners) about sexuality or sexual history with men.

^c^In the past 3 months.

### Primary Outcomes

**Figure 3:**
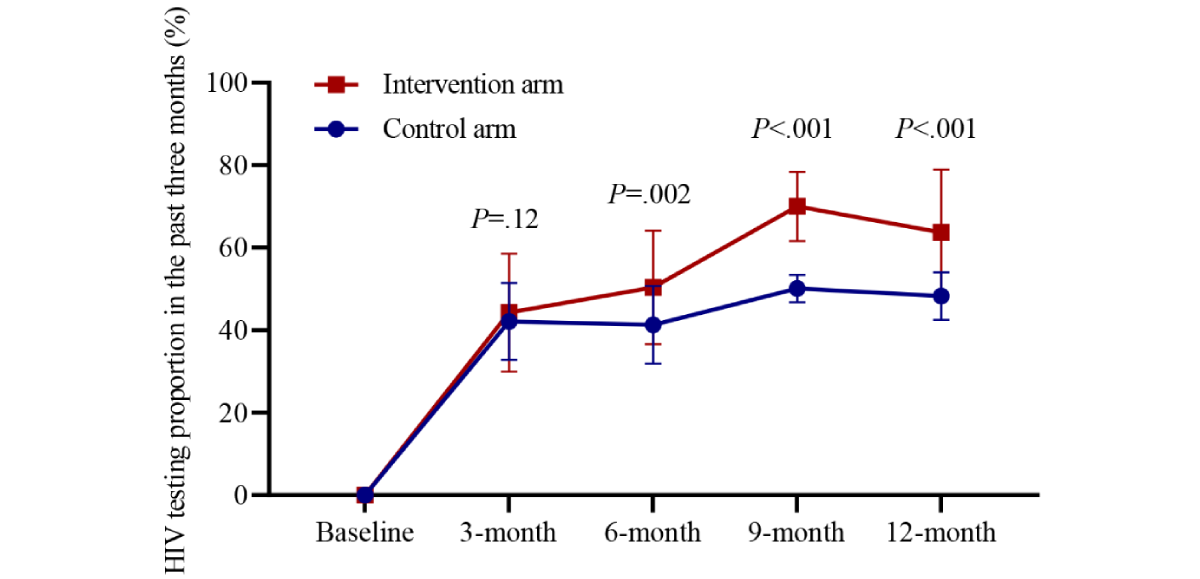
Proportion of participants who underwent HIV testing by arm at baseline and during the 4 follow-up periods. We included 751 participants who completed at least 1 of 4 follow-up surveys in this analysis.

A total of 751 participants (751/935, 80.3%) completed at least one follow-up survey and were analyzed to evaluate the effect of the intervention (Multimedia Appendix 9). Among these 751 participants, 451 (60%) reported that they were tested for HIV at least once during the follow-up period (Multimedia Appendix 10). Incident HIV testers across arms are shown in Multimedia Appendix 11. Overall, the proportion of participants testing for HIV in the past 3 months at the 3-, 6-, 9-, and 12-month follow-ups was higher in the intervention arm (139/279, 49.8%; 148/266, 55.6%; 189/263, 71.9%; and 171/266, 64.3%, respectively) than the control arm (183/418, 43.8%; 178/408, 43.6%; 206/403, 51.1%; and 182/397, 48.4%, respectively), with statistically significant differences at the 6-, 9-, and 12-month follow-ups (Figure 3, Multimedia Appendix 12).

**Figure 4:**
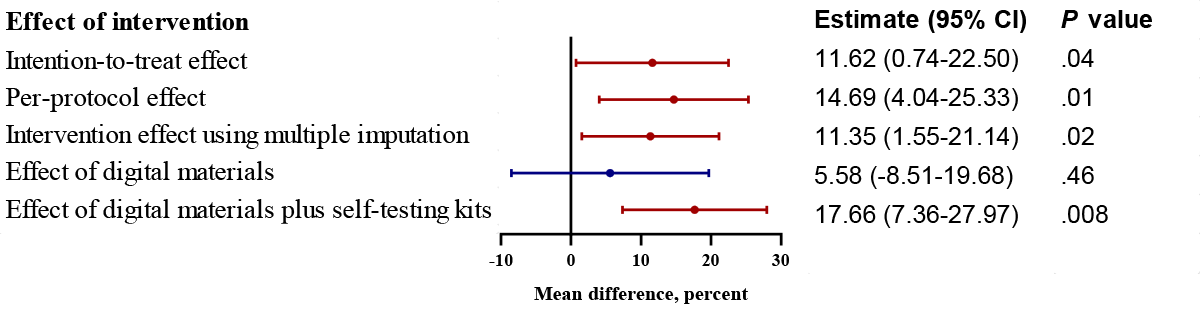
Results of a linear mixed model to determine the effect of the digital crowdsourced intervention on HIV testing uptake at the city level.

**Figure 5:**
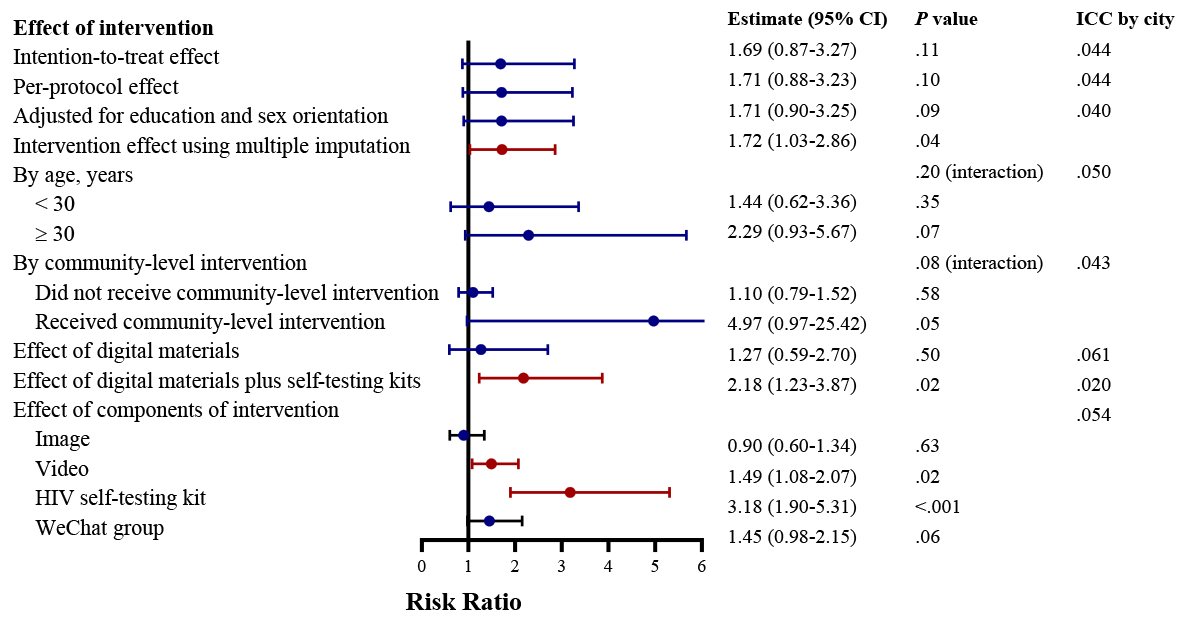
Results of a generalized linear mixed model to determine the effect of the digital crowdsourced intervention on HIV testing uptake at the individual level. ICC: interclass correlation coefficient.

For the measure of the cluster-level effect of the intervention, the proportion of individuals receiving an HIV test within a city in the intervention arm was 11.62% (95% CI 0.74%-22.50%; *P*=.04) higher than in the control arm (Figure 4). Analysis after multiple imputation produced a similar result. In the per-protocol analysis, the estimated effect size was larger (mean difference 14.69%, 95% CI 4.04%-25.33%; *P*=.01). For the individual-level effect of the intervention, results from the intention-to-treat analysis showed that participants in the intervention arm had 69% higher odds for testing for HIV in the previous 3 months compared with control participants, but the result was not statistically significant (RR 1.69, 95% CI 0.87-3.27; *P*=.11). Per-protocol analysis and sensitivity analysis adjusting for education level and sexual orientation showed similar results. An interaction was found between the individual-level intervention and the community-level intervention, but this was not statistically significant (interaction test *P*=.08) (Figure 5).

In addition, the estimated effect of the provision of digital materials alone was not statistically significant (mean difference 5.58%, 95% CI –8.51% to 19.68%; *P*=.46; RR 1.27, 95% CI 0.59-2.70; *P*=.50). After the provision of digital materials plus HIV self-testing kits, the probability that an individual had tested for HIV in the previous 3 months increased (mean difference 17.66%, 95% CI 7.36%-27.97%; *P*=.008; RR 2.18, 95% CI 1.23-3.87; *P*=.02) (Figure 4). As for the components of intervention, viewing intervention videos (RR 1.49, 95% CI 1.08-2.07; *P*=.02) and participating in peer-moderated discussion WeChat groups (RR 3.18, 95% CI 1.90-5.31; *P*<.001) increased the HIV testing uptake. A total of 287 HIV self-testing kits were applied and used by participants during the study period (Multimedia Appendix 13).

The subgroup analysis showed that the effect of the intervention was similar among MSM who were aged 18 to 30 years (RR 1.44, 95% CI 0.62-3.36) compared to MSM who were aged more than 30 years (RR 2.29, 95% CI 0.93-5.67; interaction test *P*=.20) (Figure 5).

### Secondary Outcomes

The digital, crowdsourced, multilevel intervention did not improve the secondary outcomes (Multimedia Appendix 14).

## Discussion

### Principal Findings

Despite significant achievements in HIV prevention worldwide, HIV testing uptake among MSM is still suboptimal in China. It is crucial to develop effective interventions to improve HIV testing uptake among MSM. In this cluster randomized controlled trial among Chinese MSM, the digital, crowdsourced, multilevel intervention was able to effectively enlarge HIV testing coverage, with an 11.62% increase in the proportion of HIV testing in the intervention arm. Our study extends previous research on HIV prevention interventions by using crowdsourced methods to develop tailored interventions, focusing on digital methods for intervention promotion and dissemination and addressing both individual- and community-level factors related to HIV testing.

Our findings are consistent with a previous cluster randomized controlled trial among MSM in China, which found that a crowdsourced intervention increased the number of individuals receiving an HIV test by 8.9% [23]. In addition to its use of a crowdsourced approach, this trial extends previous studies by implementing individual- and community-level interventions through digital platforms simultaneously. The fact that Chinese MSM widely use social media, mobile phone apps, and the internet to find sexual partners and search for HIV prevention information provides a basis for digital interventions. Digital tools could allow MSM to access HIV information and services without disclosing their sexual orientation, which might decrease stigma when seeking services and improve community engagement. Our findings confirm previous studies demonstrating the feasibility and effectiveness of digital-based intervention in improving HIV testing among MSM [8,20]. This trial could guide digital health intervention organization and promotion in HIV prevention programs and even other health care programs.

Most previous HIV prevention programs implemented interventions addressing factors related to HIV testing at a single level [27,28], and few studies evaluated the effect of multilevel intervention in improving HIV testing. Although there is scarce evidence on the effects of multilevel intervention for HIV testing among MSM, it has been shown to be effective in reducing sexual risk behaviors and increasing access to antiretroviral treatment among other key populations [20,29]. In this study, according to the socioecological theory [19], we developed a multilevel intervention addressing individual- and community-level factors related to HIV testing. The individual-level intervention included digital educational materials (eg, images and videos) and free HIV self-testing kits with instructions for how to use them and interpret the test results provided through WeChat, which could increase knowledge, training, and support for HIV testing services from the individual perspective. The community-level intervention included peer-led, moderated discussion and communication through WeChat groups, as well as messages shared through WeChat moments, through which MSM could obtain peer education and community support. Our findings suggest that multilevel intervention is an effective tool to expand HIV testing among MSM, highlighting the need for more multilevel interventions to promote HIV testing or other health services among key populations.

We also found that providing HIV self-testing services through digital tools effectively improved the probability of individuals receiving an HIV test. HIV self-testing services are an effective and cost-saving tool for promoting HIV testing among MSM [30,31]. Previous studies also showed the feasibility of digital HIV self-testing services and the potential effect on promoting early diagnosis in MSM [32]. Recently, the WHO has called for evidence in digital innovations to enhance HIV self-testing coverage [33]. This study provides evidence on digital HIV self-testing services to improve HIV testing among MSM in China. The digital approach might be a useful tool to make HIV self-services more available, convenient, and discreet.

### Limitations

Our study is subject to several limitations. First, the data on recent HIV testing uptake and results were self-reported by participants, which may cause information bias or recall bias. Second, sampling using digital approaches cannot reach people who do not use digital tools frequently. However, offline recruitment and peer recommendation were also used as auxiliary methods, which might alleviate this problem to some extent. Third, 28.3% (n=262) of participants were lost at the last follow-up. We performed a sensitivity analysis adjusting for factors that differed between participants lost to last follow-up and those who finished the last follow-up or seroconverted, and we used multiple imputation to examine the intervention effect. We found the results did not change. Fourth, participants in the intervention arm and the control arm came from different cities in Shandong province. However, the study cities were pair-matched according to their populations, socioeconomic contexts, and HIV burden, which could balance the participants in the 2 arms. Fifth, participants from the intervention and control cities might communicate with each other, resulting in contamination. We conducted per-protocol analyses to measure whether our results were affected by contamination and found the results did not change.

### Conclusions

This digital, crowdsourced, multilevel intervention is an effective approach to improve HIV testing uptake among MSM. Our findings highlight that such interventions should be made more widely available for HIV prevention and other public health issues.

## Appendix

Multimedia Appendix 1 CONSORT-EHEALTH checklist (V.1.6.1).

Multimedia Appendix 2 Study setting. The control arm had 5 cities: Jinan, Weifang, Zibo, Jining, and Liaocheng. The intervention arm had 6 cities: Qingdao, Dezhou, Weihai, Binzhou, Heze, and Zaozhuang. Heze and Zaozhuang are in the same cluster.

Multimedia Appendix 3 Randomization and masking.

Multimedia Appendix 4 Digital, crowdsourced, multilevel intervention and schedule.

Multimedia Appendix 5 Trial profile of each cluster.

Multimedia Appendix 6 HIV seroconversion across 4 follow-up periods.

Multimedia Appendix 7 Follow-up across 2 arms.

Multimedia Appendix 8 Baseline characteristics of study participants stratified by loss to follow-up.

Multimedia Appendix 9 Cumulative numbers of follow-up surveys completed.

Multimedia Appendix 10 HIV testing frequency over 4 follow-up periods.

Multimedia Appendix 11 Incident HIV testers in each follow-up period.

Multimedia Appendix 12 HIV testing proportion by arm over 4 follow-up periods.

Multimedia Appendix 13 Uptake of HIV self-testing kits.

Multimedia Appendix 14 Effect of digital crowdsourced intervention on secondary outcomes.

## Abbreviations

CBO: community-based organization
CDC: center for disease prevention and control
CONSORT: Consolidated Standards of Reporting Trials
GLMM: generalized linear mixed model
LMM: linear mixed model
MSM: men who have sex with men
RR: risk ratio
WHO: World Health Organization

## Data Availability

The data sets generated during and/or analyzed during this study are available from the corresponding author on reasonable request.
