# Supplementary figures and images for: Digital, Crowdsourced, Multilevel Intervention to Promote HIV Testing Among Men Who Have Sex With Men: Cluster Randomized Controlled Trial
Source: J Med Internet Res. 2023 Oct 30;25:e46890. doi: 10.2196/46890 (PMC10644183; doi:10.2196/46890)

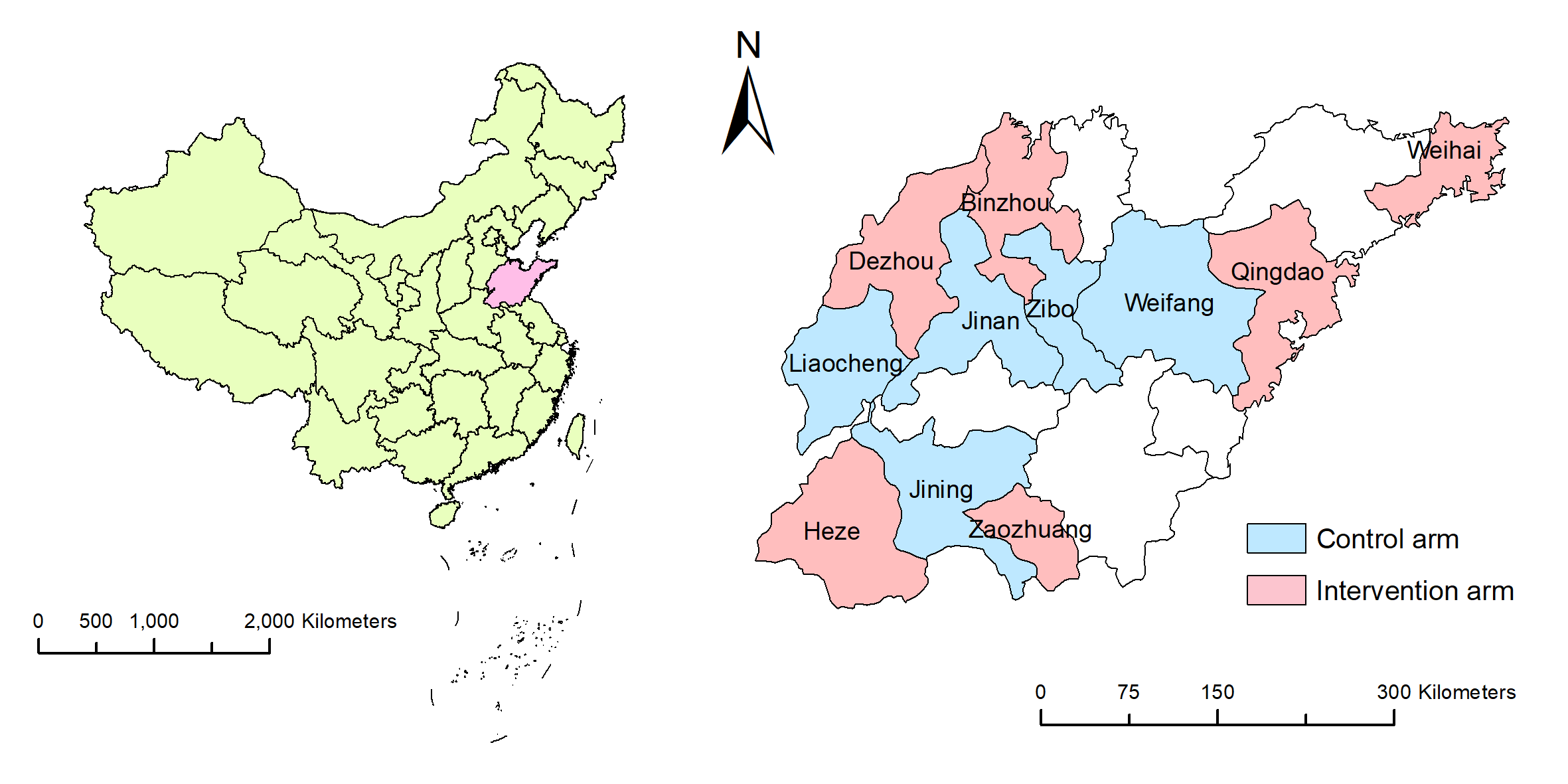

Supplement: Multimedia Appendix 2 [file jmir_v25i1e46890_app2.png]

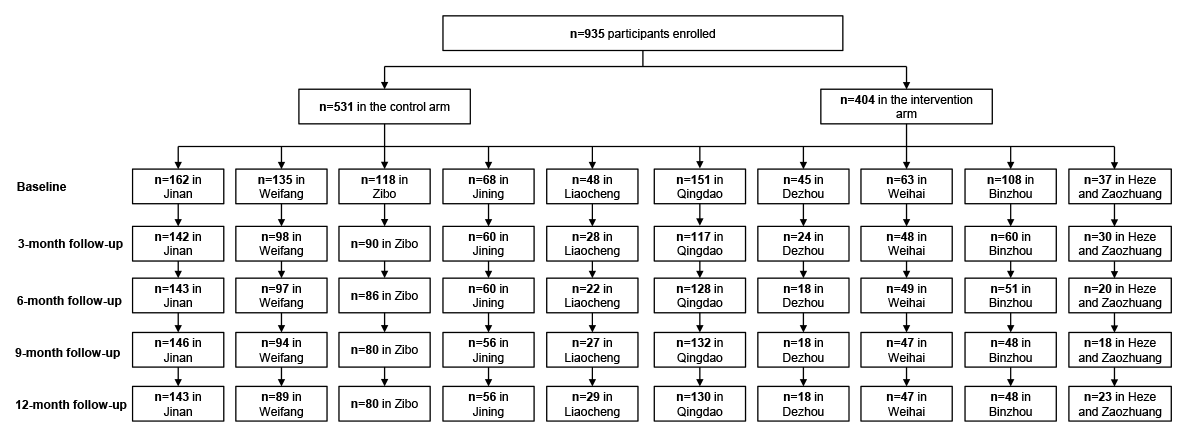

Supplement: Multimedia Appendix 5 [file jmir_v25i1e46890_app5.png]

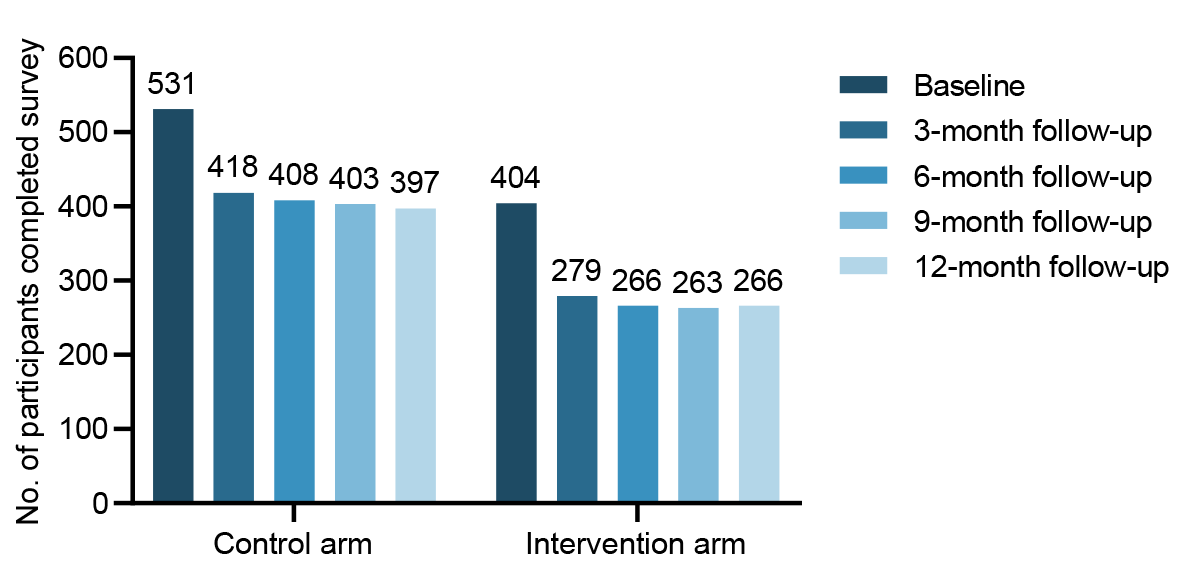

Supplement: Multimedia Appendix 7 [file jmir_v25i1e46890_app7.png]
