# Supplementary material for: Digital, Crowdsourced, Multilevel Intervention to Promote HIV Testing Among Men Who Have Sex With Men: Cluster Randomized Controlled Trial
Source: J Med Internet Res. 2023 Oct 30;25:e46890. doi: 10.2196/46890 (PMC10644183; doi:10.2196/46890)
Supplement: Multimedia Appendix 3 [file jmir_v25i1e46890_app3.docx]

# Randomization and masking

According to the city’s number of people living with HIV in 2018, average GDP in 2017, cumulative number of HIV-positive people from 2012 to 2018 and population at the end of 2017, cities with most similar population, economic, and HIV burden contexts were stratified into the same block. Ten clusters were stratified to five blocks. Block 1 consists of Jinan and Qingdao; Block 2 consists of Weifang and Dezhou; Block 3 consists of Liaocheng, Heze, and Zaozhuang; Block 4 consists of Zibo and Weihai; and Block 5 consists of Jining and Binzhou. Then two clusters in each block were randomly allocated 1:1 to either the intervention arm or the control arm with the random numbers generated using SAS 9.4 software. The intervention arm consisted of Qingdao, Dezhou, Weihai, Binzhou, Heze, and Zaozhuang. The control arm consisted of Jinan, Weifang, Zibo, Jining, and Liaocheng. Participants and data analyst were blinded to the intervention assignment.
