# Supplementary material for: Digital, Crowdsourced, Multilevel Intervention to Promote HIV Testing Among Men Who Have Sex With Men: Cluster Randomized Controlled Trial
Source: J Med Internet Res. 2023 Oct 30;25:e46890. doi: 10.2196/46890 (PMC10644183; doi:10.2196/46890)
Supplement: Multimedia Appendix 4 [file jmir_v25i1e46890_app4.docx]

**Digital, crowdsourced, multilevel intervention and schedule**

Participants in the intervention arm received digital, crowdsourced, multilevel intervention (the individual and the community-level intervention), and participants in the control arm received routine intervention by local centers for disease control and prevention (CDCs).

The individual-level intervention included the intervention materials (25 images promoting HIV testing, four videos promoting safe sex in total) and the strategy of providing HIV self-testing services through digital tools, which were developed through a series of crowdsourcing open calls conducted among MSM. The images were first showed at the end of the baseline survey, and then delivered to participants in the intervention arm biweekly via WeChat. The videos were showed at the end of each follow-up survey to the participants in the intervention arm. After completing the 6-month and 9-month follow-up surveys, participants in the intervention arm received the HIV self-testing services through WeChat, including the free HIV blood self-test kits, usage instruction, and counseling services through WeChat. First, the importance of regular testing and the process of applying for a free HIV self-testing kit were introduced to participants. Those participants who were interested in the kits provided informed consent and their addresses to get the free HIV blood self-test kits by express delivery. The instruction for using the kit were send to participants. Among those who completed the test, they were provided counseling services through WeChat and reminders to return photos of test results. The staff would help them to properly interpret the meaning and implications of the results, and provide counseling about safety sexual behaviors and regular testing for participants with negative results, and referral services on confirmatory testing and further care for participants with positive results. After the 12-month follow-up, we provided participants in both the intervention arm and the control arm with the opportunity to obtain the self-test kits to evaluate the acceptance of the kits among MSM.

| **HIV self-testing counseling services** | |
| --- | --- |
| Pre-test counseling | Post-test counseling |
| - The importance of regular testing - Process of applying for a free HIV self-testing kit - Instruction for using the HIV self-testing kit (both in video and textual form) - Informed consent form | - Interpretation of test results - Counseling services on safety sexual behaviors and regular testing for participants with negative results - Referral services on confirmatory testing and further care for participants with positive results |

For the community-level intervention, we set up eight WeChat-groups in the intervention group (each cluster had a single group, except that Qingdao had four groups due to the large number of participants). All participants in the intervention arm were invited to these groups and determined to participant by themselves. Each WeChat-group consisted of one team member, one volunteer of CBOs, and 20-30 participants. The messages about HIV testing and safety sexual behavior from authoritative facilities (CDCs, CBOs, hospitals, etc.) were shared through WeChat-groups and WeChat-moments (a function on WeChat that people can share their life with friends) biweekly. In addition, we also encouraged members in the WeChat-groups to ask questions about HIV prevention and discuss them with each other.

Due to the impact of COVID-19, the intervention was postponed until Mar 31, 2020, when the epidemic of COVID-19 had been widely controlled in Shandong Province, China. Follow-up surveys were conducted every three months thereafter for 12 months.

| **Schedule** | **Intervention arm** | | | | | **Control arm** | |
| --- | --- | --- | --- | --- | --- | --- | --- |
|  | Individual-level intervention | | | Community-level intervention | | HIV self-testing kits | Routine intervention by local CDCs |
|  | Images | Videos | HIV self-testing kits | Messages shared through WeChat-groups and We-Chat moments | Discussion in WeChat-groups |  |  |
| Intervention period | √ |  |  | √ | Any time |  |  |
| 3-mo follow-up |  | √ |  |  |  |  |  |
| Intervention period | √ |  |  | √ |  |  |  |
| 6-mo follow-up |  | √ |  |  |  |  |  |
| Intervention period | √ |  | √ | √ |  |  |  |
| 9-mo follow-up |  | √ |  |  |  |  |  |
| Intervention period | √ |  | √ | √ |  |  |  |
| 12-mo follow-up |  | √ |  |  |  |  |  |
| After study |  |  | √ |  |  | √ |  |
