# Supplementary material for: Digital, Crowdsourced, Multilevel Intervention to Promote HIV Testing Among Men Who Have Sex With Men: Cluster Randomized Controlled Trial
Source: J Med Internet Res. 2023 Oct 30;25:e46890. doi: 10.2196/46890 (PMC10644183; doi:10.2196/46890)
Supplement: Multimedia Appendix 6 [file jmir_v25i1e46890_app6.docx]

# HIV seroconversion across 4 follow-up periods

| **Time** | **Percent, (No. participants who were HIV seroconverted/No. participants who completed survey)** | | |
| --- | --- | --- | --- |
|  | **Control arm** | **Intervention arm** | **Total** |
| 3-month | 0.7 (3/418) | 1.1 (3/279) | 0.9 (6/697) |
| 6-month | 0.5 (2/408) | 0.8 (2/266) | 0.6 (4/674) |
| 9-month | 0.0 (0/403) | 0.0 (0/263) | 0.0 (0/666) |
| 12-month | 1.5 (6/397) | 0.8 (2/266) | 1.2 (8/663) |
