# Supplementary material for: Digital, Crowdsourced, Multilevel Intervention to Promote HIV Testing Among Men Who Have Sex With Men: Cluster Randomized Controlled Trial
Source: J Med Internet Res. 2023 Oct 30;25:e46890. doi: 10.2196/46890 (PMC10644183; doi:10.2196/46890)
Supplement: Multimedia Appendix 8 [file jmir_v25i1e46890_app8.docx]

# Baseline characteristics of study participants stratified by loss to follow-up

| **Characteristic** | **No. (%)** | | ***P* value** |
| --- | --- | --- | --- |
|  | **Lost to follow-up (*n* = 262) ^a^** | **Completed last follow-up survey or seroconverted ^b^ (*n* = 673)** |  |
| Age, years | | | |
| < 30 | 170 (64.9) | 431 (64.0) | .81 |
| ≥ 30 | 92 (35.1) | 242 (36.0) |  |
| Time of living in cities | | | |
| ≤ 2 years | 19 (7.3) | 60 (8.9) | .41 |
| > 2 years | 243 (92.7) | 613 (91.1) |  |
| Marital status | | | |
| Never married | 187 (71.4) | 494 (73.4) | .82 |
| Married | 51 (19.5) | 121 (18.0) |  |
| Divorced | 24 (9.2) | 58 (8.6) |  |
| Monthly income, USD ^c^ | | | |
| < 250 | 32 (12.2) | 111 (16.5) | .19 |
| 251-500 | 44 (16.8) | 95 (14.1) |  |
| 501-800 | 133 (50.8) | 308 (45.8) |  |
| 801-1 250 | 43 (16.4) | 118 (17.5) |  |
| > 1 250 | 10 (3.8) | 41 (6.1) |  |
| Educational degree | | | |
| High school or lower | 107 (40.8) | 199 (29.6) | .001 |
| College or higher | 155 (59.2) | 474 (70.4) |  |
| Sexual orientation | | | |
| Homosexuality | 168 (64.1) | 500 (74.3) | .002 |
| Other | 94 (35.9) | 173 (25.7) |  |
| Disclosure of sexual orientation | | | |
| Not disclosed to others | 105 (40.1) | 224 (33.3) | .05 |
| Disclosed to others ^d^ | 157 (59.9) | 449 (66.7) |  |
| Condomless sex ^e^ | | | |
| No | 185 (70.6) | 436 (64.8) | .09 |
| Yes | 77 (29.4) | 237 (35.2) |  |
| Ever tested for HIV | | | |
| No | 47 (17.9) | 103 (15.3) | .32 |
| Yes | 215 (82.1) | 570 (84.7) |  |

^a^ Lost to follow-up was defined as missed the last follow-up survey.

^b^ 10 participants seroconverted before the 12-mo follow-up.

^c^ 1 USD= 6 CNY in 2021.

^d^ Has told anyone (except sexual partners) about sexuality or sexual history with men.

^e^ In the past three months.
