# Supplementary material for: Digital, Crowdsourced, Multilevel Intervention to Promote HIV Testing Among Men Who Have Sex With Men: Cluster Randomized Controlled Trial
Source: J Med Internet Res. 2023 Oct 30;25:e46890. doi: 10.2196/46890 (PMC10644183; doi:10.2196/46890)
Supplement: Multimedia Appendix 9 [file jmir_v25i1e46890_app9.docx]

# Cumulative numbers of follow-up surveys completed

| **No. of follow-up surveys participants completed** | **No. of participants** |
| --- | --- |
| 1 | 47 |
| 2 | 45 |
| 3 | 63 |
| 4 | 596 |
| Total | 751 |
