# Supplementary material for: Digital, Crowdsourced, Multilevel Intervention to Promote HIV Testing Among Men Who Have Sex With Men: Cluster Randomized Controlled Trial
Source: J Med Internet Res. 2023 Oct 30;25:e46890. doi: 10.2196/46890 (PMC10644183; doi:10.2196/46890)
Supplement: Multimedia Appendix 10 [file jmir_v25i1e46890_app10.docx]

# HIV testing frequency over 4 follow-up periods

| **No. of follow-up periods during which participant reported HIV testing** | **No.** | | |
| --- | --- | --- | --- |
|  | **Total (n=451)** | **Control arm (n=255)** | **Intervention arm (n=196)** |
| 1 | 130 | 80 | 50 |
| 2 | 79 | 45 | 34 |
| 3 | 82 | 44 | 38 |
| 4 | 160 | 86 | 74 |
