# Supplementary material for: Digital, Crowdsourced, Multilevel Intervention to Promote HIV Testing Among Men Who Have Sex With Men: Cluster Randomized Controlled Trial
Source: J Med Internet Res. 2023 Oct 30;25:e46890. doi: 10.2196/46890 (PMC10644183; doi:10.2196/46890)
Supplement: Multimedia Appendix 11 [file jmir_v25i1e46890_app11.docx]

# Incident HIV testers in each follow-up period

| **Arm** | **Participants who completed at least one follow-up survey, No.** | **Incident HIV testers in each follow-up period ^a^** | | | | |
| --- | --- | --- | --- | --- | --- | --- |
|  |  | **3-month, No.** | **6-month, No.** | **9-month, No.** | **12-month, No.** | **Total, No. (%)** |
| Control | 445 | 183 | 1 | 46 | 25 | 255 (57.3%) |
| Intervention | 306 | 139 | 0 | 44 | 13 | 196 (64.1%) |
| **Arm** | **Participants who completed at least one follow-up survey, No.** | **Incident HIV testers (among participants who had never tested for HIV at baseline) in each follow-up period ^b^** | | | | |
|  |  | **3-month, No.** | **6-month, No.** | **9-month, No.** | **12-month, No.** | **Total, No. (%)** |
| Control | 61 | 15 | 0 | 8 | 2 | 25 (41.0%) |
| Intervention | 53 | 16 | 0 | 4 | 4 | 24 (45.2%) |

^a^ We included 751 participants who completed at least one of four follow-up surveys in this analysis.

^b^ We included 114 participants who completed at least one of four follow-up surveys and have never tested for HIV at baseline in this analysis.
