# Supplementary material for: Digital, Crowdsourced, Multilevel Intervention to Promote HIV Testing Among Men Who Have Sex With Men: Cluster Randomized Controlled Trial
Source: J Med Internet Res. 2023 Oct 30;25:e46890. doi: 10.2196/46890 (PMC10644183; doi:10.2196/46890)
Supplement: Multimedia Appendix 12 [file jmir_v25i1e46890_app12.docx]

# HIV testing proportion by arm over 4 follow-up periods

| **Time** | **HIV testing proportion in the past three months, percent (No. participants tested/No. total participants)** | | ***P* value** |
| --- | --- | --- | --- |
|  | **Control arm** | **Intervention arm** |  |
| Enrolment, n | 531 | 404 |  |
| 3-month | 43.8 (183/418) | 49.8 (139/279) | .12 |
| 6-month | 43.6 (178/408) | 55.6 (148/266) | .002 |
| 9-month | 51.1 (206/403) | 71.9 (189/263) | <.001 |
| 12-month | 48.4 (192/397) | 64.3 (171/266) | <.001 |
