# Supplementary material for: Digital, Crowdsourced, Multilevel Intervention to Promote HIV Testing Among Men Who Have Sex With Men: Cluster Randomized Controlled Trial
Source: J Med Internet Res. 2023 Oct 30;25:e46890. doi: 10.2196/46890 (PMC10644183; doi:10.2196/46890)
Supplement: Multimedia Appendix 13 [file jmir_v25i1e46890_app13.docx]

# Uptake of HIV self-testing kits

| **Time** | **No. (%)** | | | | | | | | |
| --- | --- | --- | --- | --- | --- | --- | --- | --- | --- |
|  | **Control arm** | | | **Intervention arm** | | | **Total** | | |
|  | **Received kits** | **Returned results** | **Positive results** | **Received kits** | **Returned results** | **Positive results** | **Received kits** | **Returned results** | **Positive results** |
| After 6-month | NA | NA | NA | 88 (33.1) | 73 (83.0) | 0 (0.0) | 88 (33.1) | 73 (83.0) | 0 (0.0) |
| After 9-month | NA | NA | NA | 69 (26.2) | 43 (62.39) | 0 (0.0) | 69 (26.2) | 43 (62.39) | 0 (0.0) |
| After 12-month | 87 (21.9) | 69 (71.1) | 3 (4.3) | 43 (16.2) | 25 (58.1) | 0 (0.0) | 130 (19.6) | 94 (72.3) | 3 (3.2) |
| Total | 87 (21.9) | 69 (71.1) | 3 (4.3) | 200 (25.2) | 141 (70.5) | 0 (0.0) | 287 (24.1) | 210 (73.2) | 3 (1.4) |

A total 210 participants returned their self-testing kit results (207 negative results and 3 positive results), 208 (99.0%) self-testing kit results matched self-reported results. 207 participants who reported having not tested for HIV or receiving negative HIV testing results in the past three months returned negative self-testing kit results. One participant who reported receiving positive HIV testing results in the past three months returned positive self-testing kit results. Two participants who reported having not tested for HIV in the past three months returned positive self-testing kit result.
