# Supplementary material for: Digital, Crowdsourced, Multilevel Intervention to Promote HIV Testing Among Men Who Have Sex With Men: Cluster Randomized Controlled Trial
Source: J Med Internet Res. 2023 Oct 30;25:e46890. doi: 10.2196/46890 (PMC10644183; doi:10.2196/46890)
Supplement: Multimedia Appendix 14 [file jmir_v25i1e46890_app14.docx]

# Effect of digital crowdsourced intervention on secondary outcomes

| **Secondary outcomes** | **Estimate (95% CI)** | ***P* value** | **ICC by city** |
| --- | --- | --- | --- |
| Risk ratio | | | |
| HIV facility-based testing | 1.39 (0.63-3.04) | .37 | .064 |
| HIV self-testing | 1.14 (0.69-1.86) | .56 | .015 |
| Frequent of HIV testing | 0.84 (0.46-1.53) | .52 | .031 |
| Number of sexual partners | 1.04 (0.62-1.72) | .87 | .018 |
| Frequency of anal sex | 0.95 (0.54-1.66) | .84 | .022 |
| Condomless sex | 1.33 (0.97-1.82) | .07 | <.001 |
| Social media engagement | 1.14 (0.60-2.16) | .65 | .036 |
| Mean difference | | | |
| Anticipated HIV stigma | -0.13 (-1.16-0.89) | .80 | .106 |
| HIV testing social norm | -0.22 (-0.69-0.25) | .36 | .020 |
| HIV testing self-efficacy | 0.14 (-0.83-1.11) | .77 | .084 |

Abbreviations: CI, confidence interval; ICC, interclass correlation coefficient.
